# Rapid laser ablation-based fabrication of high-density polymer microwell arrays for high-throughput cellular studies†

**DOI:** 10.1039/d4lc01058b

**Published:** 2025-02-27

**Authors:** Desh Deepak Dixit, Kavya L. Singampalli, Amit S. Niyogi, Amanda Montoya, Alexandre Reuben, Peter B. Lillehoj

**Affiliations:** a Department of Mechanical Engineering, Rice University Houston TX USA lillehoj@rice.edu; b Medical Scientist Training Program, Baylor College of Medicine Houston TX USA; c Department of Bioengineering, Rice University Houston TX USA; d Department of Thoracic/Head and Neck Medical Oncology, Division of Cancer Medicine, The University of Texas MD Anderson Cancer Center Houston TX USA

## Abstract

Polymer-based microwell platforms have garnered much interest due to their usefulness in culturing and analyzing small quantities of biological cells and spheroids. Existing methods for fabricating polymer microwell arrays involve complex fabrication processes and/or are limited in their ability to create dense arrays of very small (<50 μm in diameter) microwells. Here, we present a simple and rapid technique for fabricating high-density arrays of microwells ranging from 20 to 160 μm in diameter on a variety of polymer substrates. In this approach, a polymer surface is ablated using a CO_2_ laser that is rastered over a stainless steel mesh, which serves as a shadow mask. A theoretical laser–polymer interaction model was developed for predicting the microwell volume based on the substrate properties and laser settings. Microwell volumes predicted by the model were within 5.4% of fabricated microwell volumes determined experimentally. Cellulose acetate microwell arrays fabricated using this technique were used to culture Lewis lung carcinoma cells expressing ovalbumin (LLC-OVA), which were maintained for up to 72 h with a negligible (<5%) loss in viability. As a second proof of principle demonstration, LLC-OVA cells grown in microwell arrays were co-cultured with OT-I T cells and measurements of interferon gamma (IFN-γ), a marker for T cell activation, were performed which revealed a positive correlation between LLC-OVA cell-T cell interaction time and T cell activation. These two *in vitro* demonstrations showcase the capability of this technique in generating polymer microwell arrays for high-throughput cellular studies, including cell growth dynamics studies and cell interaction studies. Furthermore, we envision that these platforms can be used with different cell types and for other biological applications, such as spheroid formation and single cell analysis, further expanding the utility of this technique.

## Abstract 2

We report a simple laser ablation-based technique for the rapid fabrication of high-density polymer microwell arrays for high-throughput cellular applications, including cell growth dynamics studies and cell interaction studies.

## Graphical abstract

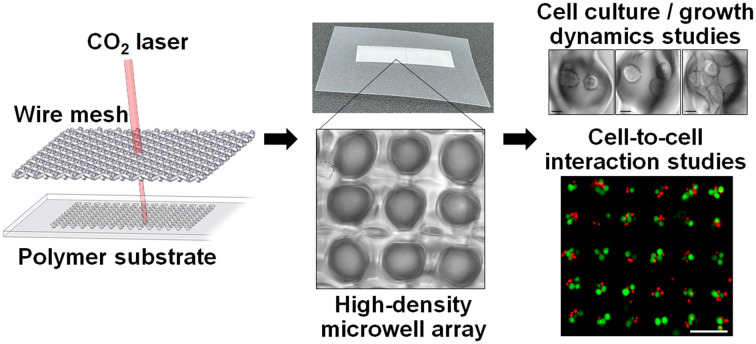

## Introduction

Cell culture and cell analysis traditionally rely on the use of flat, plastic cell/tissue culture dishes, which are affordable, versatile and offer excellent biocompatibility. However, the use of such platforms yields a continuous layer of cells making it extremely difficult to obtain individual cell responses.^1–5^ To address this limitation, microwell-based platforms have emerged as a valuable tool for culturing and analyzing single and small populations of cells. These platforms typically consist of an array of microwells ranging in size from 10's to 1000's of microns, enabling each well to contain between one and a few hundred cells. Microwells have been used in various cellular applications ranging from single cell profiling,^6^ cell expansion,^7^ co-culture^8^ and response studies,^9,10^ formation of organoids,^11,12^ and tissue engineering.^13^ Depending on the application, the microwell size is an important parameter. For example, studies involving the 3D aggregation of cells, such as the formation of multicellular tumor spheroids^14,15^ and organ-on-chip platforms,^16^ require microwells that are 100's to 1000's of microns in diameter to accommodate the size of the cell aggregates. Larger microwells also facilitate long-term experiments aimed at studying proliferation dynamics by providing adequate space for cell growth and expansion.^17^ Conversely, studies involving single or few cells require smaller-sized microwells that are typically <100 μm in diameter,^18,19^ which allow more sensitive/consistent measurements of cell activity or response due to the confinement of the cells within a small volume.^20^

The fabrication of polymer microwell arrays can be achieved using a variety of fabrication techniques. Conventional microfabrication techniques^21–27^ can be used to fabricate microwells ranging from a few microns to a few hundred microns in diameter.^28^ However, these techniques rely on photolithography, which is laborious, time-consuming and needs to be performed in a cleanroom. Furthermore, microwells fabricated using photolithography are typically made with UV-sensitive photoresists, many of which exhibit moderate biocompatibility, making them less desirable for long-term experiments.^29^ Soft lithography (replica molding) offers similar versatility as photolithography in regards to the microwell sizes that can be fabricated, while offering higher throughput,^21,30^ however, it requires the fabrication of a master mold and is limited to elastomeric materials. Techniques involving chemical etching can also produce microwells with a broad range of sizes,^31^ however, they require a chemically-inert mask that is fabricated using complicated techniques (*e.g.*, photolithography) or highly specific etchants that are incompatible with some polymer substrates.

Alternative methods for creating polymer microwell arrays include micro-milling/drilling,^26^ inkjet printing^32^ and 3D printing,^33^ which do not require the fabrication of a separate mask or mold, or the use of a cleanroom. However, these methods require specialized (and costly) equipment, are limited to specific types of materials and typically offer low throughput. Laser ablation has also been employed for the fabrication of polymer microwell arrays,^11,34–38^ which is relatively simple to perform and offers low-cost operation and high compatibility with a broad range of polymer substrates.^37^ However, existing laser ablation-based methods are not amenable to large-scale production since microwells are fabricated one at a time and/or are unable to create very small microwells (<50 μm diameter) using consumer-grade laser cutting/engraving systems.

Here, we report a laser ablation-based technique for the rapid fabrication of polymer microwells with diameters ranging from 20 μm to 160 μm. This method employs a unique approach in which a CO_2_ laser is rastered over a stainless steel mesh, which serves as a shadow mask. The polymer surface exposed to laser beam is ablated while the areas covered by the mesh remain intact, resulting in the formation of microwells. Using this approach, we were able to fabricate high-density (up to ∼17 700 wells per cm^−2^) microwell arrays over an area of 10 mm × 35 mm within 20 s. Microwell arrays were fabricated on various polymer substrates, including cellulose acetate, polymethyl methacrylate (PMMA), polyethylene terephthalate (PET), cyclic olefin polymer (COP), polymerized SU-8 photoresist and polydimethylsiloxane (PDMS), which all exhibit high optical transparency, excellent biocompatibility and are commonly used for culturing and analyzing biological cells.^39–47^ We also show that the microwell size can be tuned by adjusting the laser settings (power and rastering speed) using a theoretical laser–polymer interaction model, which is validated through experimental measurements of fabricated microwells. As a proof of principle demonstration, Lewis lung carcinoma cells expressing ovalbumin (LLC-OVA) were cultured in cellulose acetate microwell arrays and were maintained for 72 h with a negligible (<5%) loss in viability. To further demonstrate the utility of microwell arrays generated using this technique for cellular analysis, LLC-OVA cells were grown in cellulose acetate microwells and co-cultured with OT-I T cells. Measurements of interferon gamma (IFN-γ), a marker for T cell activation, were performed which revealed a positive correlation between LLC-OVA cell-T cell interaction time and T cell activation, showcasing the utility of laser-ablated microwell arrays for cell interaction studies.

## Materials and methods

### Microwell fabrication

Microwells were fabricated on polymer substrates using a 60 W CO_2_ laser system (Universal Laser System) (Fig. S1a†). A stainless steel wire mesh was attached to the substrate using a custom fixture (Fig. S1b†). Meshes with different mesh sizes were used, including a 325 × 325 mesh (McMaster-Carr #9319 T188), a 200 × 200 mesh (McMaster-Carr #9319 T191), a 150 × 150 mesh (McMaster-Carr #9319 T185) and a 80 × 80 mesh (McMaster-Carr #9319 T181). A 200 μm-thick PMMA spacer was placed between the mesh and the substrate to prevent them from adhering to each other during laser ablation (Fig. S1c†). Microwell arrays were fabricated on various materials, including PET (thickness = 120 μm, McMaster-Carr #8567 K54), PMMA (thickness = 250 μm, McMaster-Carr #4076 N13), cellulose acetate (thickness = 100 μm, Optiazure), PDMS (Sylgard 184, Dow), polymerized SU-8 (Kayaku Advanced Materials #Y111069T) and COP (thickness = 140 μm, Zeon - Zeonor ZF14 Film). The polymerized SU-8 substrate was fabricated by spin coating (Laurell Technologies) SU-8 onto PET followed by exposure to UV light (365 nm) for 1.5 min. The PDMS substrate was fabricated by combining Sylgard 184 elastomer base and curing agent at a 10 : 1 ratio (by weight), followed by mixing and degassing in a vacuum chamber for 30 min. The degassed PDMS mixture was spin coated onto PET and cured in a convection oven at 80 °C for 2 h. The thickness of the SU-8 and PDMS substrates was ∼100 μm. Microwells with different dimensions were generated by varying the laser power and rastering speed.

### Characterization of microwells

Microwells were imaged using a digital microscope (VHX-S7000, Keyence). Microwell dimensions were obtained using the Keyence measurement software. For each combination of laser power and rastering speed that was used to fabricate microwells, measurements were performed on three unique samples at three randomly selected locations on each sample. At each of the three locations, the microwell diameter and depth were measured 10 times. Cross-sectional profiles of the microwells were obtained using a Dektak XT stylus profilometer (Bruker) with a 2 μm stylus radius. Measurements were performed with a stylus force of 3 mg and a scan rate of 33.3 μm s^−1^.

### Cell culture

LLC cells were obtained from American Type Cell Collection and transduced to endogenously express the H-2Kb-restricted OVA257-264 antigen and cultured using Roswell Park Memorial Institute (RPMI) 1640 complete cell culture medium (Corning 10 040 CV) supplemented with 10% fetal bovine serum (FBS) (referred to as “complete RPMI media” hereafter). Selection was maintained in transduced LLC-OVA cells by adding 0.05 mg mL^−1^ of hygromycin B (Corning #30-240-CR) to the cell culture media. To measure H-2Kb presentation of the OVA257-264 antigen on LLC-OVA cells, a PE anti-H-2Kb bound to OVA257-264 antibody (clone 25-D1.16, Biolegend #141604) was added to the target cells at 0.2 mg mL^−1^ and incubated on ice for 30 min. Samples were acquired using a Cytek Aurora full spectrum cytometer and analyzed using FlowJo™ v10.10.0 software (BD Biosciences), gating on single cells.

### Murine OT-I CD8+ T cell isolation and expansion

All animal procedures were approved by the MD Anderson Institutional Animal Care and Use Committee and conducted in accordance with its Guidelines for Care and Use of Laboratory Animals. Female transgenic OT-I mice (C57BL/6-Tg (TcraTcrb)1100Mjb/J; RRID:IMSR_JAX:003831), aged 8–12 weeks at the time of sacrifice, were used for the study. Spleens were isolated and disrupted in complete RPMI media, after which the cell suspensions were passed through a 40 μm nylon mesh strainer (Fisher Scientific #22363547) to remove debris and aggregates. The strained suspensions were then centrifuged at 600 g for 5 min at room temperature to pellet the cells.

A red cell lysis buffer (Santa Cruz Biotechnology #sc-296258) was prepared at a 1× concentration in sterile Millipore water and added to the pelleted cells at a volume 10× that of the pellet. Following a 12 min incubation at room temperature, complete RPMI media was added at 10× the volume of the lysis buffer to quench the reaction. The cells were then centrifuged again at 600 g for 6 min, resuspended in biotin, Ca^2+^, and Mg^2+^-free 1× PBS supplemented with 2% FBS and 1 mM ethylenediaminetetraacetic acid, at a concentration of ≤1 × 10^8^ nucleated cells per mL. Negative CD8+ selection was then used to isolate CD8+ splenocytes *via* magnetic sorting under sterile conditions using the STEMCELL Technologies' EasySepTM mouse CD8+ isolation kit (catalog #19853A) per manufacturer's instructions. Isolated CD8+ T cells were counted using VitaStain™ AOPI staining solution (Nexcelom Biosciences #CS2-0106) and a Nexcelom Cellometer® K2 fluorescent viability cell counter. Isolated CD8+ T cells were activated using Dynabeads™ Mouse T-Activator CD3/CD28 (Thermofisher Scientific #11452D) and cultured for 7 days using complete RPMI media supplemented with 100 U mL^−1^ of Penicillin–Streptomycin (Thermofisher Scientific #15140122) and 100 IU mL^−1^ of IL-2 (Stem Cell Technologies #78036). After 7 days, the beads were removed from the culture and CD8+ OT-I T cells were cryopreserved in FBS with 10% dimethyl sulfoxide.

### Immunofluorescence staining

LLC-OVA cells were detached using 0.25% trypsin (2.21 mM, Corning #25-053), and washed once with 10× the volume of complete RPMI media, followed by a wash with 1× PBS. The cells were then resuspended in 1× PBS at a concentration of ≤5 × 10^5^ cells per mL and stained with 1 μM of carboxyfluorescein succinimidyl ester (CFSE, Selleckchem #S8269) for 30 min at room temperature. After staining, an equal volume of complete RPMI media was added, and the cells were washed again. Finally, the cells were resuspended in complete RPMI media before being added to the microwells.

OT-I CD8+ T cells were thawed and washed with complete RPMI media, followed by a wash with 1× PBS. The cells were then resuspended in 1× PBS at a concentration of ≤5 × 10^5^ cells per mL and stained with 0.5 μM of CellTracker Deep Red (Invitrogen #134565A) for 30 min at 37 °C. After staining, an equal volume of complete RPMI media was added, and the cells were washed again. Finally, the cells were resuspended in complete RPMI media before being added to the microwells.

### Cell growth experiments

A microwell array (well diameter = 60 μm) fabricated on cellulose acetate was adhered to the bottom of a 35 mm petri dish (Corning, #351008) using PDMS. The microwell array was treated with O_2_ plasma (Plasma Etch) for 30 s for sterilization and to enhance the surface wettability. LLC-OVA cells were seeded into the microwells by pipetting ∼300 000 cells into the array and scraping the surface to ensure that the cells had filled the microwells. Cells were incubated with complete RPMI media at 37 °C and 5% CO_2_ with the media being changed daily. After varying incubation times ranging from 24–72 h, three microwell arrays per incubation time were imaged to quantify cell growth. Microwells with at least one cell were included in the count and at least 100 microwells per array were considered. Cells incubated for 72 h in the microwell array were stained with 10 μM of propidium iodide (Sigma-Aldrich #P4864) and imaged using a Zeiss Axio Imager M2 microscope to assess cell viability.

### T cell–tumor cell interaction experiments

OT-I T cell-LLC-OVA cell interaction experiments were performed in 96-well plates. A microwell array (well diameter = 60 μm) fabricated on cellulose acetate was cut into 6 mm-diameter circles and adhered to the bottom of the 96-well plate wells using PDMS. ∼10 000 LLC-OVA cells (stained with CFSE) were seeded onto each microwell array and incubated with 300 μL of complete RPMI media for 24 h. At that time, the media was removed to remove unbound cells, and ∼10 000 OT-I T cells (stained with CellTracker Red) were added to each microwell array. The LLC-OVA cells and OT-I T cells were co-incubated for up to 48 h. At 8, 24 and 48 h time points, media was removed from the wells of the 96-well plate, frozen, and stored at −80 °C. The media was tested for IFN-γ levels using a commercial IFN-γ ELISA kit (BioLegend #430804) following the manufacturer's instructions. Measurements of IFN-γ levels were also performed on media sampled from wells incubated with OT-I T cells only at the same time points.

## Results & discussion

### Microwell fabrication principle and microwell characterization

**Fig. 1:**
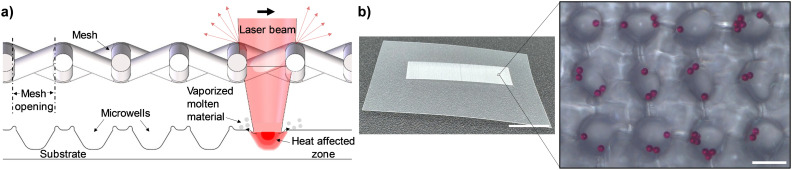
Principle of the laser ablation-based microwell fabrication technique. (a) Schematic depicting the process of generating microwell arrays in a polymer substrate by rastering a CO_2_ laser over a stainless steel mesh. (b) Photograph of a high-density (∼17 700 microwells per cm^−2^) microwell array fabricated over a 10 mm × 35 mm area on a 100 μm-thick cellulose acetate substrate. Scale bar, 10 mm. Inset shows a magnified view of the microwells seeded with 10 μm-diameter red polystyrene beads. Scale bar, 50 μm.

In contrast to existing laser ablation-based microwell fabrication methods where microwells are generated one at a time, our technique allows for the fabrication of high-density microwell arrays by rastering a CO_2_ laser over a stainless steel mesh, which is positioned above the polymer substrate. The polymer surface exposed to laser beam is ablated while the areas covered by the mesh remain intact, resulting in the formation of microwells (Fig. 1a). During the laser ablation process, the laser energy is absorbed by the polymer substrate, which locally heats the surface causing it to melt and vaporize the polymer.^48^ The micron-sized openings in the mesh (Fig. S1d†) reduces the laser spot size by partially blocking/scattering the laser beam, which enables the creation of very small microwells. Furthermore, high-density microwell arrays (up to ∼17 700 wells per cm^−2^) could be produced over an area of up to 10 mm × 35 mm (Fig. 1b) in less than 20 s, which cannot be achieved using existing fabrication methods. Initially, the mesh was placed in direct contact with the substrate, which was found to leave an imprint or fuse with the substrate following the laser ablation process. Therefore, a 200 μm-thick PMMA spacer was used to maintain a constant gap between the mesh and the substrate, ensuring uniform formation of microwells. The shape of the microwell opening is circular with a slight elongation along one axis, which we attribute to the laser beam being slightly elliptical in shape, resulting in an asymmetric Gaussian laser energy profile.^36,38^ There is a small hump-shaped protrusion around the outer rim of the microwell opening, resulting from the accumulation of molten polymer being ejected from the ablation site.^49^ This observation is consistent with prior studies that show the presence of protruding structures around the opening of polymer microwells fabricated *via* laser ablation.^37,38^ The hump-shaped protrusions are located outside of the microwells and should not cause any adverse effects on cells cultured within the microwells. Two-dimensional profilometry measurements of the microwells revealed them having a cone-shaped cross-sectional profile with smooth interior surfaces (Fig. S2†).

**Fig. 2:**
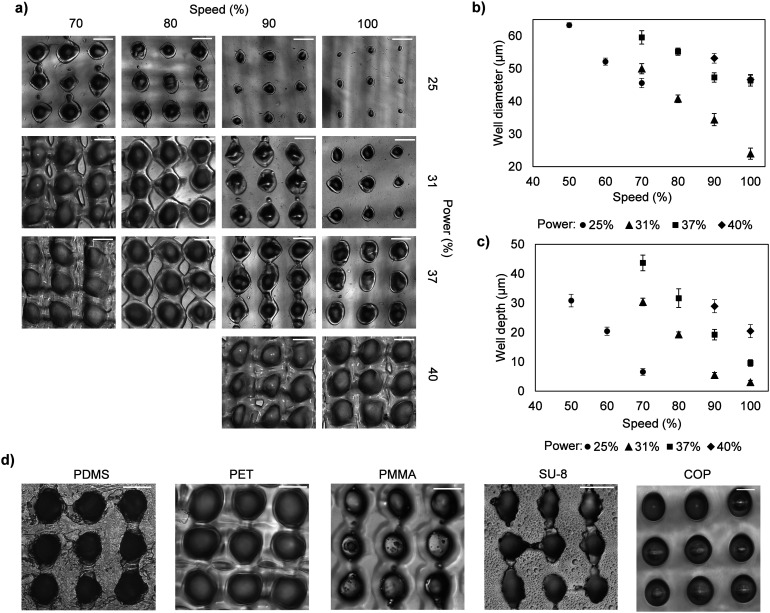
Characterization of microwells. (a) Optical micrographs of microwells in cellulose acetate fabricated using a 325 × 325 mesh with different combinations of laser power and rastering speed. Scale bars, 50 μm. Plots of laser power and rastering speed *vs.* (b) microwell diameter, and (c) microwell depth. Each point represents the mean ± standard deviation (SD) of 30 independent measurements. (d) Optical micrographs of microwells in PDMS, PET, PMMA and SU-8 fabricated using a 325 × 325 mesh, and microwells in COP fabricated using 200 × 200 mesh. Scale bars, 50 μm.

To demonstrate the capability of generating microwells with different dimensions, microwell arrays were fabricated on various polymer substrates using a 325 × 325 mesh with different combinations of laser power and laser rastering speed. A laser power of 100% corresponds to 60 W and a rastering speed of 100% corresponds a laser beam velocity of 1.27 m s^−1^. When these settings are reduced to 25% power and 70% speed, this corresponds to a laser power of 15 W and laser velocity of 0.89 m s^−1^, respectively. A higher laser power results in a greater amount of material being ablated from the surface due to an increased amount of energy that is applied to the surface. Similarly, a slower rastering speed results in greater material ablation due to the surface being exposed to the laser for a longer amount of time. Thus, adjusting the laser power and/or rastering speed alters the material removal rate, which results in the formation of different-sized microwells. A parametric study of the influence of the laser power and rastering speed on the microwell dimensions was performed using a cellulose acetate substrate. We observed that 25% power was the minimum laser power needed to generate microwells (at the highest rastering speed of 100%), whereas the use of >40% power caused excessive ablation (resulting in no microwell formation) at speeds <80%. Therefore, 25% to 40% power and 70% to 100% speed were selected as the optimal ranges for generating microwells in cellulose acetate. We observed a positive correlation between the laser power/rastering speed and the microwell opening size (Fig. 2a). For a specific rastering speed, higher laser powers resulted in the formation of larger-diameter and deeper microwells (Fig. 2b, c and S2†). Likewise, slower rastering speeds resulted in the formation of larger-sized microwells for a specific laser power.

Microwell arrays were also fabricated using meshes with larger mesh sizes, including a 200 × 200 mesh (opening size = 74 μm), a 150 × 150 mesh (opening size = 104 μm) and a 80 × 80 mesh (opening size = 152 μm) with 15% power and 100% speed. Larger meshes (*i.e.*, those with larger mesh openings) generated larger microwells with diameters ranging from 110 μm to 160 μm (Fig. S3†). Similar to the 325 × 325 mesh, high-density microwell arrays with narrow spacing between the microwells were generated using the 200 × 200 and 150 × 150 meshes. Microwells fabricated using the 80 × 80 mesh were spaced further apart due to the mesh opening size being larger than the laser spot size (diameter ≈ 130 μm). These results demonstrate the versatility of this technique in being able to quickly generate dense arrays of larger microwells, which can be useful for applications involving the growth or analysis of biological spheroids. Microwell arrays were fabricated in other polymer substrates, including PDMS, PET, PMMA, COP and SU-8, further demonstrating the versatility of this technique (Fig. 2d). Microwells generated in these materials exhibited a similar geometry as those generated in cellulose acetate with some minor differences. The PDMS substrate surface exhibited crack-like patterns around the microwell openings which we attribute to the thermal oxidative degradation of PDMS resulting from laser ablation.^50,51^ Similarly, the openings of the SU-8 microwells had irregular edges and there was residue on the substrate surface which we attribute to the carbonization of SU-8 due to the laser ablation process.^52,53^

### Theoretical modeling

**Fig. 3:**
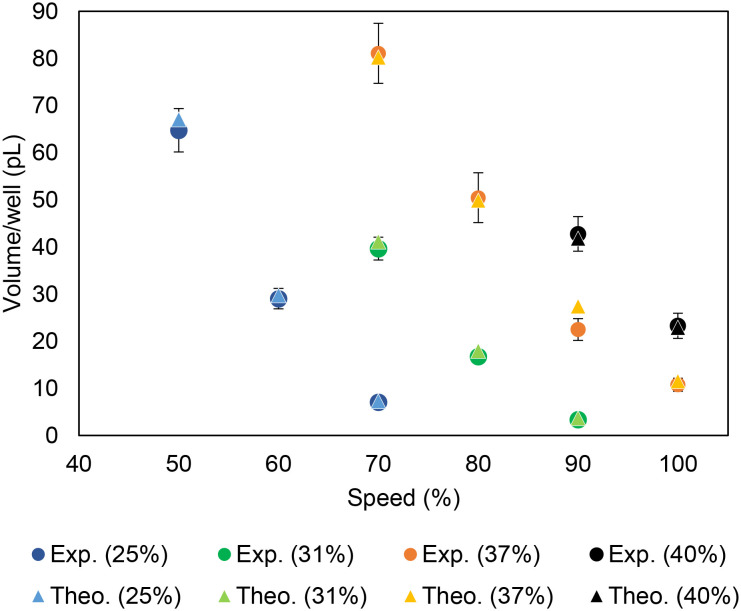
Theoretical microwell volume predicted by the model *vs.* experimental volume measurements of cellulose acetate microwells fabricated using a 325 × 325 mesh with different laser powers (denoted by different colored data points) and rastering speeds. Each point represents the mean ± SD of 30 independent measurements.

We developed a theoretical laser–polymer interaction model which could predict the microwell volume based on the substrate properties and laser settings. The theoretical ablated mass was estimated using a laser ablation model (additional details about this model are presented in ESI†). In this model, laser energy absorbed by the substrate heats up the laser spot on the surface, causing the polymer to melt and vaporize. This model considers multiple laser parameters (laser fluence, spot size, energy) and material parameters (thermal diffusivity, density, fluence of material) to estimate the ablated mass (eq. 1–4 in ESI†). Microwell volumes predicted by the model were well correlated (<5.4% difference) with volumes of fabricated microwells determined from experimental measurements of microwell dimensions for a broad range of laser powers and rastering speeds (Fig. 3). These results validate the accuracy of the model in predicting the microwell size, which can facilitate the design of microwell arrays for applications requiring specific microwell dimensions.

### Cell growth experiments

**Fig. 4:**
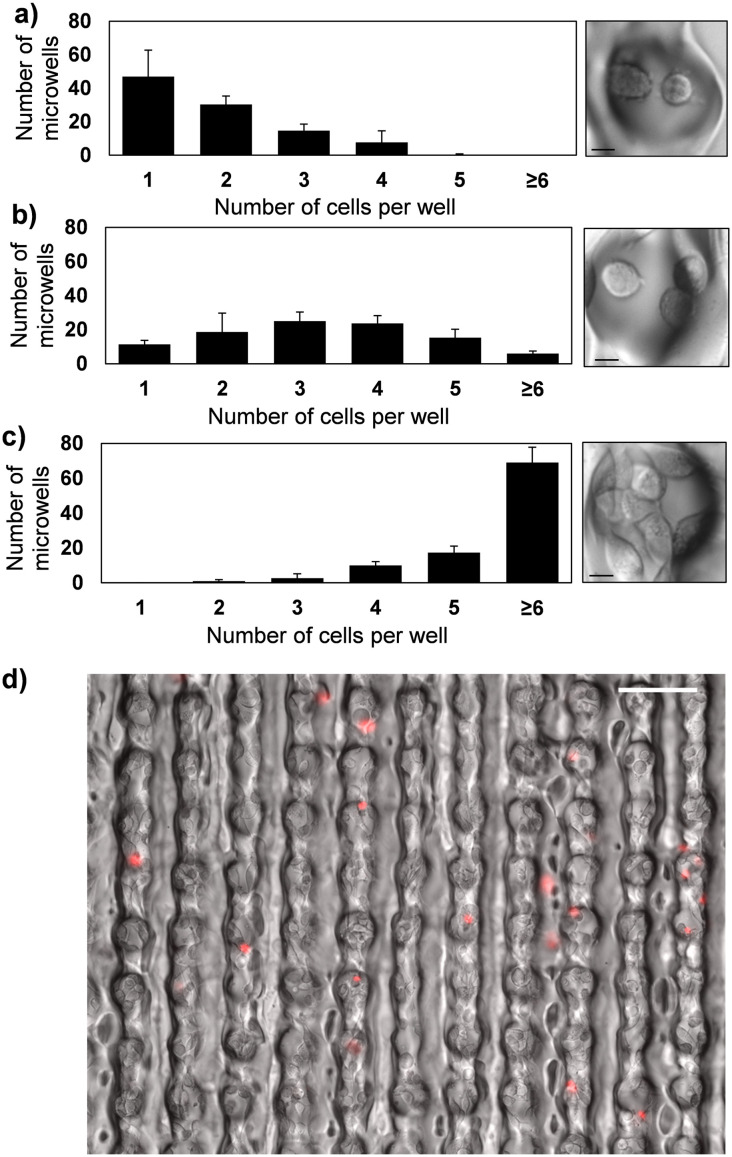
Growth of LLC-OVA cells in cellulose acetate microwells. Histograms showing the number of microwells with 1, 2, 3, 4, 5, or ≥ 6 cells at (a) 24 h, (b) 48 h, and (c) 72 h. Each bar represents the mean ± SD of measurements from three microwell arrays. Optical micrographs depict representative microwells with the average number of cells per microwell at each time point. Scale bars, 10 μm. (d) Representative optical micrograph of LLC-OVA cells stained with propidium iodide after 72 h of culture in the microarray array. Scale bar, 100 μm.

A unique feature of this fabrication technique is its ability to generate high-density arrays of miniature microwells having diameters as small as 20 μm, enabling a small number of cells (*i.e.*, 1–3) to be contained within each microwell, making it useful for monitoring cell proliferation for growth dynamics studies. However, one challenge associated with using small microwells in aqueous environments is that air bubbles can become trapped in the microwells due to their high surface tension compared to the polymer surface, which is mildly hydrophobic. To prevent this issue, the microwell arrays were treated with an O_2_ plasma to enhance their wettability, enabling the cell suspension to spread evenly over the microwell array and minimizing the likelihood of trapped air bubbles. To demonstrate the utility of microwell arrays fabricated using this technique for cell culture and growth dynamics studies, LLC-OVA cells were cultured in cellulose acetate microwell arrays having a well diameter of 60 μm. Due to the small size of each microwell, >77% of the wells contained 1–2 cells after 24 h of being seeded into the array. The majority of the cells adhered to the inside of the microwells within 24 h, as would be expected with traditional cell culture plastic (Fig. 4a). After 48 h, the number of cells per microwell increased, with >67% of the wells containing 2–4 cells, indicating robust cell growth (Fig. 4b). At 72 h, the cells continued to proliferate within the microwells and >86% of wells contained >4 cells (Fig. 4c). Additionally, the LLC-OVA cells maintained >95% viability after growing inside the microwells for 72 h (Fig. 4d). These collective results indicate that microwells fabricated using this technique are suitable for culturing small population of cells, making this approach promising for cell growth studies.

### T cell–tumor cell interaction experiments

**Fig. 5:**
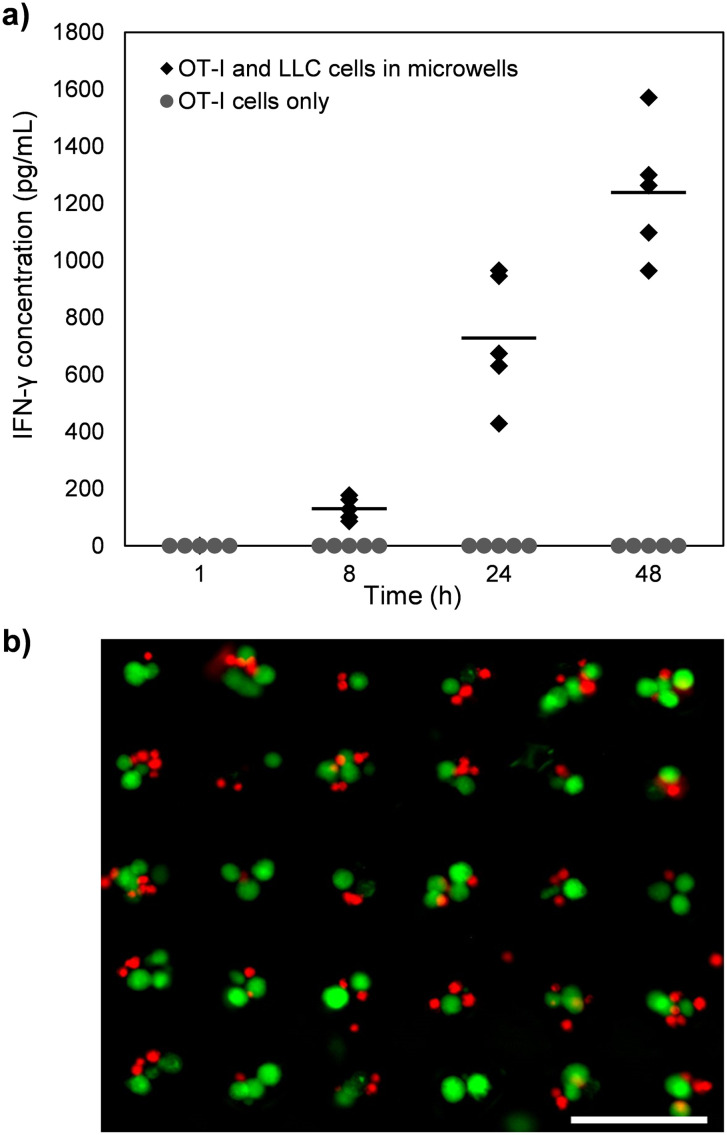
Activation of OT-I T cells after interaction with LLC-OVA cells. (a) Absorbance values produced by an IFN-γ ELISA for culture media of OT-I T cells co-incubated with LLC-OVA cells in microwells for 1 to 48 h and of OT-I T cells only. Each data point represents an independent measurement (*n* = 5). (b) Representative fluorescent image of LLC-OVA cells (green) and OT-I T cells (red) co-cultured in a cellulose acetate microwell array. Scale bar, 100 μm.

To further demonstrate the utility of microwell arrays fabricated using this technique, LLC-OVA cells grown in cellulose acetate microwells integrated within a 96-well plate were co-cultured with OT-I T cells to study their interaction and evaluate T cell activation. Measurements of cytokines, such as IFNs, tumor necrosis factors and interleukins, secreted by T cells has shown to be a strong indicator for T cell activation.^54^ Therefore, we evaluated T cell activation by measuring the amount of IFN-γ in the culture media obtained from the wells of the 96-well plate at various time points. We confirmed the expression of SIINFEKL antigen on the LLC-OVA cells by staining them with the SIINFEKL-specific PE antibody and analyzing the cells using flow cytometry. This analysis confirmed that 90% of viable single LLC-OVA cells express the SIINFEKL antigen (Fig. S4†). Further, we observed a positive correlation between the LLC-OVA cell-OT-I T cell incubation time and the concentration of IFN-γ in the media (Fig. 5a), indicating robust activation of the OT-I T cells due to sustained interactions with the LLC-OVA cells. These results are consistent with prior studies showing an increase in OT-I T cell activation in the presence of OVA antigen.^55–57^ In contrast, levels of IFN-γ remained negligible for the media containing OT-I T cells only for the duration of the experiment. One advantage of using small microwells for T cell activation studies is that each microwell contains at least ∼1–2 tumor cells, which increases the likelihood of a T cell interacting with one or more tumor cells, resulting in the secretion of elevated amounts of IFN-γ, which can increase the assay sensitivity. Furthermore, the small size of the microwells enables the OT-I T cells and the LLC-OVA cells to maintain close proximity throughout the experiment (Fig. 5B), facilitating cell-to-cell interactions and enhancing T cell activation.

## Conclusions

We have presented a unique laser ablation-based technique for the rapid fabrication of high-density microwell arrays with tunable dimensions ranging from 20 to 160 μm in diameter. Using this technique, microwell arrays were fabricated on various polymer substrates, including cellulose acetate, PMMA, PET, PMDS, SU-8 and COP. We demonstrate the ability to tune the microwell size simply by adjusting the laser settings, which is validated through theoretical modeling and experimental measurements of microwell dimensions fabricated using this technique. We demonstrate proof of principle by culturing LLC-OVA cells in cellulose acetate microwell arrays, which maintained >95% viability after 72 h of culture. Microwell arrays were also used to study the activation of OT-I T cells co-cultured with LLC-OVA tumor cells. These *in vitro* experiments showcase the utility of microwell arrays fabricated using this technique for cell growth studies and cell interaction studies using small populations of cells. Furthermore, the ability to perform such studies using high-density arrays makes this approach useful for high-throughput screening, drug discovery, and cancer research. We envision that this technique can be used to create microwell arrays for other biological applications, such as spheroid/organoid formation and single cell analysis, and with different cell types further expanding the utility of this technique.

## Data availability

The data supporting this article have been included as part of the ESI.† Further data are available upon reasonable request from the authors.

## Author contributions

D. D. D.: conceptualization, data curation, formal analysis, investigation, methodology, validation, visualization, writing – original draft preparation, writing – review & editing. K. L. S., A. S. N., A. M.: data curation, formal analysis, investigation, validation, visualization, writing – original draft preparation, writing – review & editing. A. R.: writing – review & editing, supervision, resources, project administration, funding acquisition. P. B. L.: formal analysis, methodology, visualization, writing – original draft preparation, visualization, writing – review & editing, supervision, resources, project administration, funding acquisition.

## Conflicts of interest

There are no conflicts to declare.

## Supplementary Material

LC-025-D4LC01058B-s001
