# Supplementary material for: Rapid laser ablation-based fabrication of high-density polymer microwell arrays for high-throughput cellular studies
Source: Lab Chip. 2025 Feb 27;25(7):1813–22. doi: 10.1039/d4lc01058b (PMC11880749; doi:10.1039/d4lc01058b)
Supplement: LC-025-D4LC01058B-s001 [file LC-025-D4LC01058B-s001.pdf]

Supplementary Information (SI) for Lab on a Chip.
This journal is © The Royal Society of Chemistry 2025

Supporting Information for

Rapid laser ablation-based fabrication of high-density polymer microwell arrays for high-throughput cellular studies

Desh Deepak Dixit[a], Kavya L. Singampalli[b,c], Amit Niyogi[a], Amanda Montoya[d], Alexandre Reuben[d], Peter B. Lillehoj[a,c*]

[a] Department of Mechanical Engineering, Rice University, Houston, TX, USA

[b] Medical Scientist Training Program, Baylor College of Medicine, Houston, TX, USA

[c] Department of Bioengineering, Rice University, USA

[d] Department of Thoracic/Head and Neck Medical Oncology, Division of Cancer Medicine, The University of Texas MD Anderson Cancer Center, Houston, TX, USA

*Corresponding author: lillehoj@rice.edu

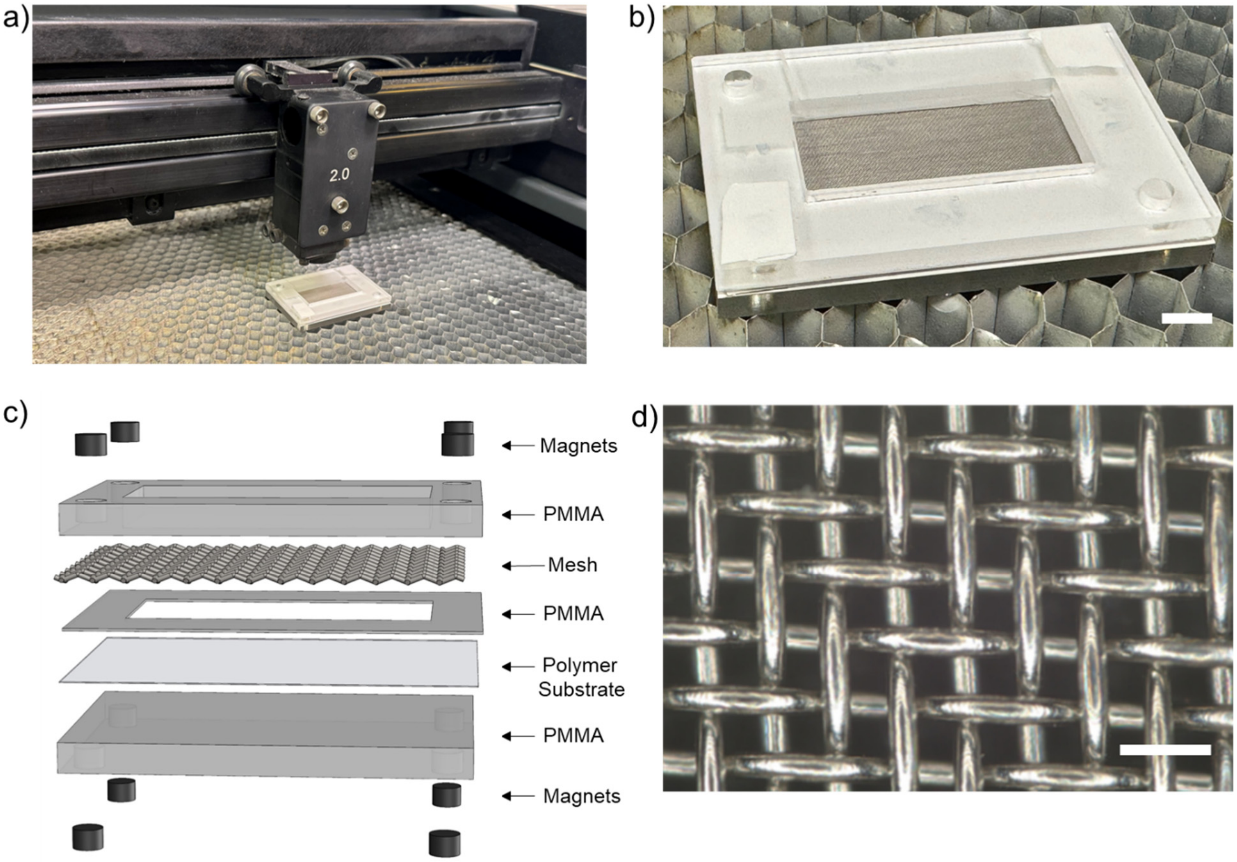

Figure S1. Overview of the experimental setup for laser ablation-based fabrication of polymer microwell arrays. (a) Photograph of the fixture placed inside the CO_2 laser cutting/engraving system. (b) Photograph showing a close-up view of the fixture. Scale bar, 5 mm. (c) Exploded view of the fixture. (d) Optical micrograph showing a close-up view of the stainless steel 325 × 325 mesh. Scale bar, 100 µm.

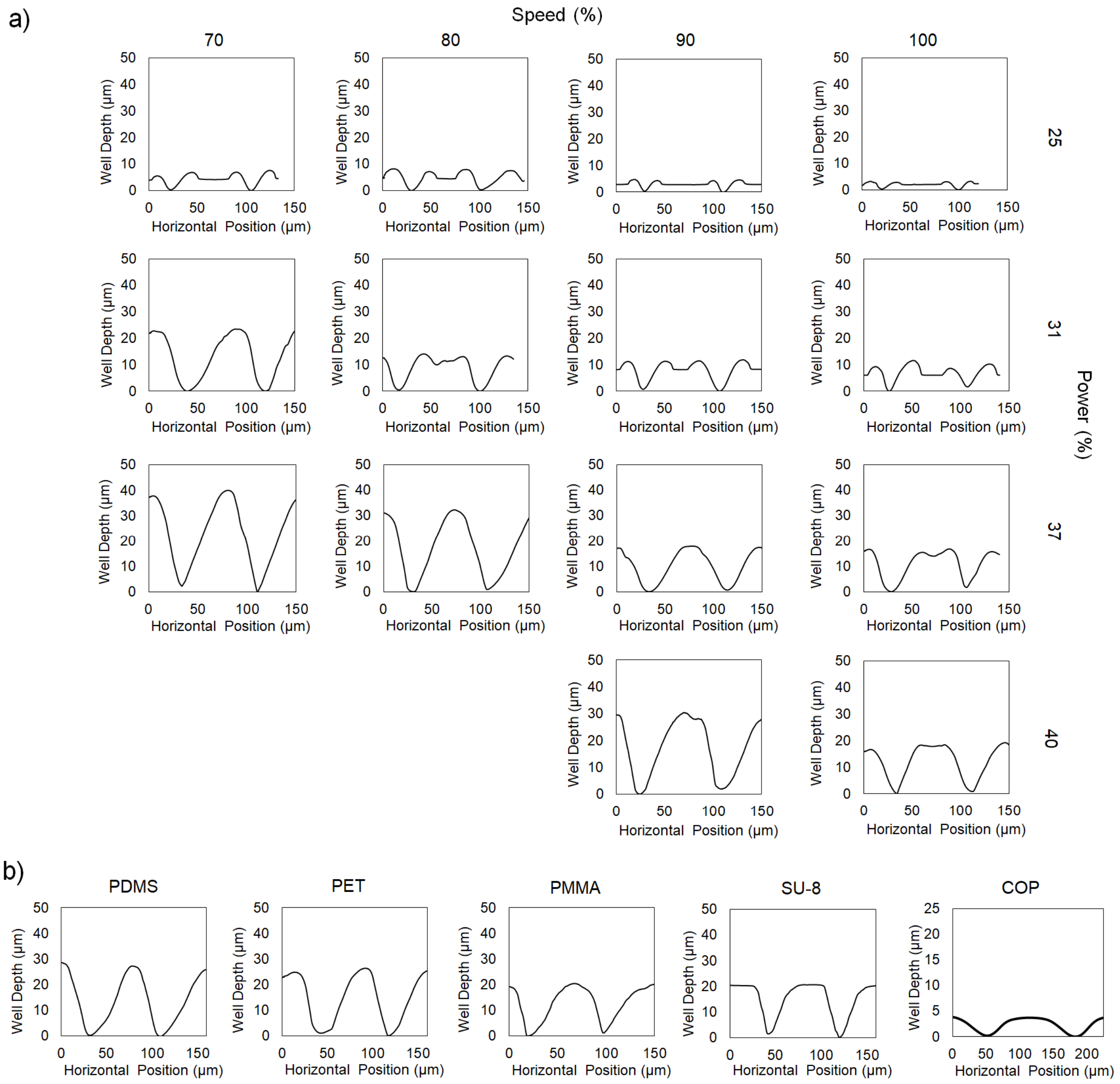

Figure S2. Cross-sectional profiles of microwells. (a) Profiles of microwells in cellulose acetate fabricated using a 325 × 325 mesh with different combinations of laser power and rastering speed. (b) Profiles of microwells in PDMS, PET, PMMA and SU-8 fabricated using a 325 × 325 mesh, and microwells in COP fabricated using a 200 × 200 mesh.

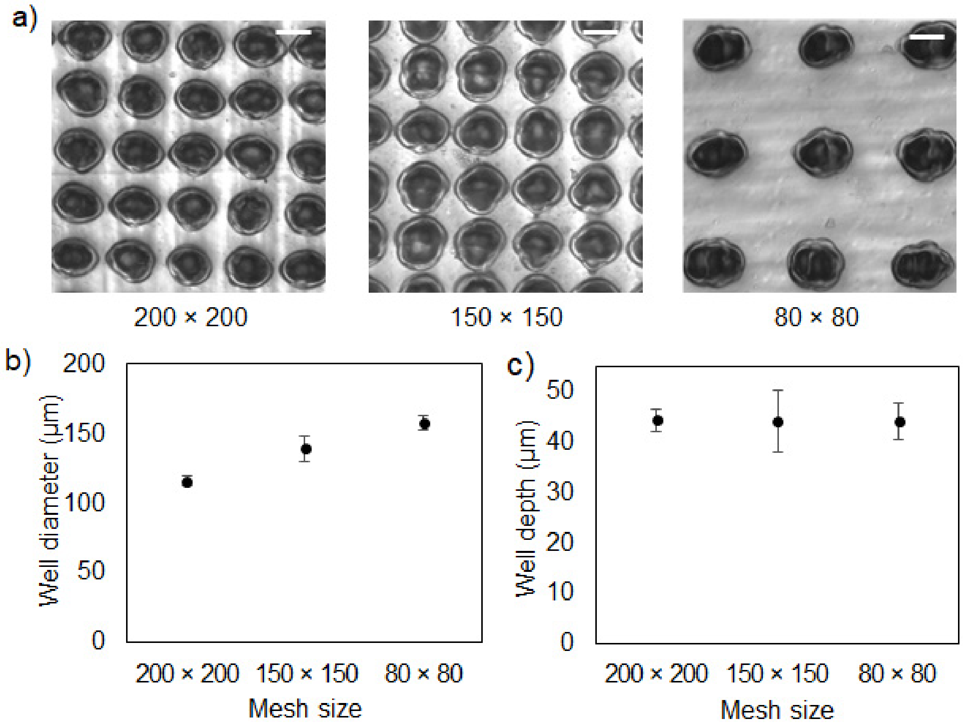

Figure S3. Characterization of microwells fabricated using meshes with larger mesh sizes. (a) Optical micrographs of cellulose acetate microwells fabricated using a 200 × 200 mesh, a 150 × 150 mesh and a 80 × 80 mesh with 15% power and 100% speed. Scale bars, 100 µm. Plots of (b) microwell diameter and (c) microwell depth for microwells fabricated with different mesh sizes. Each data point represents the mean ± SD of 30 independent measurements.

Theoretical Modeling of Microwell Volume. The ablated mass, m, was calculated using Eq. 1[1] where ρ is the density of the substrate, w_o is the spot size (i.e., the size of mesh opening), D is the thermal diffusivity, τ is the laser pulse time, φ_o is the laser fluence, φ_a is the material threshold fluence of the substrate and χ is the attenuation factor (χ = 0.5 in this work). The laser fluence was calculated using Eq. 2, where P is the maximum power of the laser, p is the fraction of laser power used for ablation, d is the mesh opening size and a is the correction factor to account for energy loss due to the scattering/blocking of the laser beam from the mesh and loss of heat to the surroundings ($a = 0.145$ in this work). The residence time of the laser, t, was calculated using Eq. 3, where s is the fraction of speed used in ablation and S is the maximum linear speed of the laser beam. The material threshold fluence was calculated using Eq. 4[2] where C is the heat capacity of the substrate and T_v is the vaporization temperature or thermal degradation temperature if the material degrades before vaporization[4]. The thermal diffusivity, D, was calculated using Eq. 5[5] where K is the thermal conductivity of the material. The values of the parameters described above are presented in Table S1.

Table S1. Parameters for modeling laser ablation of polymer and the associated values used in this work.

Parameter	Value	Reference
τ	$280\ \mu s$	equipment datasheet
w_o	$130\ \mu m$	equipment datasheet
ρ	$1300\ Kg/m^3$	6
P	$60\ W$	equipment datasheet
S	$1.27\ m/s$	equipment datasheet
T_v	$540\ K$	4
d	$50\ \mu m$	material datasheet
C	$1464\ J/kgK$	6

$$m = \frac{8\pi\rho w_o^2}{3}\sqrt{D\tau}\left(2\sqrt{2}-1\right)ln\left(\chi\frac{\varphi_o}{\varphi_a}\right) \qquad (1)$$

$$\varphi_o = a\left(\frac{pPt}{d^2}\right) \qquad (2)$$

$$t = d/sS \qquad (3)$$

$$\varphi_a = 2C\rho T_v\sqrt{\pi D t} \qquad (4)$$

$$D = K/\rho C \qquad (5)$$

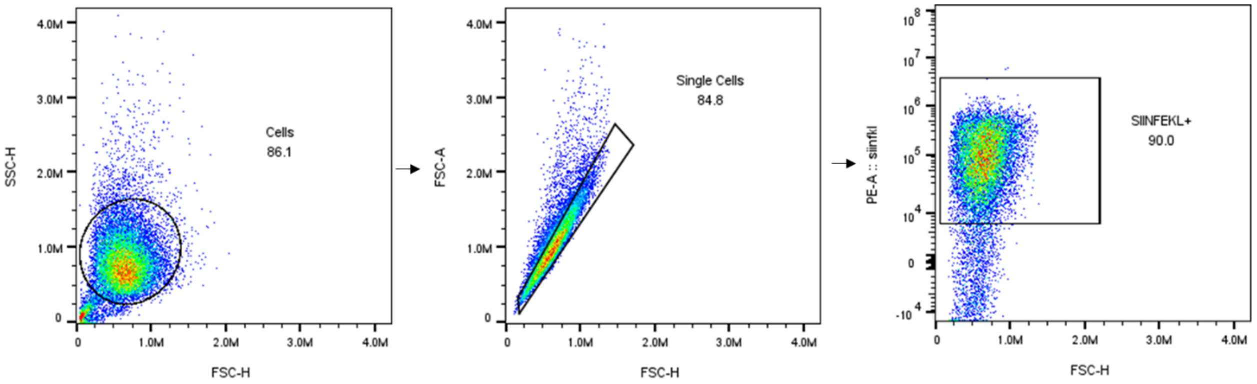

Figure S4. SIINFEKL expression in LLC cells. LLC cells were stained with SIINFEKL-specific PE antibody and analyzed using flow cytometry to determine the expression levels of the SIINFEKL antigen. SIINFEKL positive cells were gated on single cells.

References

(1) Sinko, J. E.; Phipps, C. R.; Sasoh, A. Modeling CO2 Laser Ablative Impulse with Polymers. *AIP Conf. Proc.* **2010**, *1278*, 699–709. https://doi.org/10.1063/1.3507163.

(2) Sinko, J. E.; Phipps, C. R.; Tsukiyama, Y.; Ogita, N.; Sasoh, A.; Umehara, N.; Gregory, D. A. Critical Fluences and Modeling of Co 2 Laser Ablation of Polyoxymethylene from Vaporization to the Plasma Regime. In *AIP Conference Proceedings*; 2010; Vol. 1230, pp 395–407.

(3) John E. Sinko; Claude R. Phipps; Yosuke Tsukiyama; Naoya Ogita; Akihiro Sasoh; Noritsugu Umehara; Don A. Gregory. Critical Fluences And Modeling Of CO2 Laser Ablation Polyoxymethylene From Vaporization To The Plasma Regime. *AIP Conf. Proc.* **2010**, *1230*, 395–407.

(4) Kenji KAMIDE, M. S. Thermal Analysis of Cellulose Acetate Solids with Total Degree of 0.49, 1.75, 2.46, and 2.92. *Polym. J.* **1985**, *17* (8), 919–928.

(5) Salazar, A. On Thermal Diffusivity. *Eur. J. Phys.* **2003**, *24* (4), 351–358. https://doi.org/10.1088/0143-0807/24/4/353.

(6) Verros, G. D.; Malamataris, N. A. Estimation of Diffusion Coefficients in Acetone - Cellulose Acetate Solutions. *Ind. Eng. Chem. Res.* **1999**, *38* (9), 3572–3580. https://doi.org/10.1021/ie990141d.
